# Meningitis Relapse in a Neonate: From Extended-Spectrum Beta-Lactamases-Producing Escherichia coli to Escherichia coli K1

**DOI:** 10.7759/cureus.31601

**Published:** 2022-11-17

**Authors:** Salma S Alkattan, Shams AlTurki, Basim F Khan

**Affiliations:** 1 Pediatrics, Imam Abdulrahman Bin Faisal University, Dammam, SAU; 2 Pediatrics, King Fahd University Hospital, Al-Khobar, SAU

**Keywords:** extended-spectrum beta-lactamases, preterm neonate, escherichia coli k1, escherichia coli, meningitis

## Abstract

We report the case of a 10-day-old preterm neonate with extended-spectrum beta-lactamase (ESBL)-producing *Escherichia coli *meningitis that was treated appropriately and yet relapsed with *E. coli* K1 meningitis at the age of one month. Although the patient had multiple risk factors for ESBL-producing *E. coli*, the patient did not follow the reported pattern of the risk of *E. coli* K1 infection in relapse.

## Introduction

A group of beta-lactamases known as extended-spectrum beta-lactamases (ESBLs) have the ability to evolve rapidly and hydrolyze third-generation cephalosporins and monobactams (i.e., aztreonam). The two most common bacteria that produce ESBL and cause infections, predominantly in children, are *Klebsiella pneumoniae* and *Escherichia coli *[1]. The people most infected by ESBL are healthcare workers, patients with a prolonged hospital stay, patients with a recent stay in the intensive care unit, prolonged use of antibiotics, and immunocompromised patients. Symptoms depend on the system involved, which may include the urinary tract, gastrointestinal tract, skin via wounds, or through blood (bacteremia) [2].

Furthermore, involvement of the brain has been emerging recently in the form of meningitis; however, this involvement is extremely rare [3]. *E. coli* is the most common cause of neonatal meningitis, in particular, *E. coli* with K1 strains. Moreover, there have been reported cases of *E. coli* K1 relapses [4].

As uncommon as it is for ESBL to cause meningitis, we report a case of ESBL-producing *E. coli* that was treated with an appropriate course of antibiotics, yet had a relapse with *E. coli* K1.

## Case presentation

A preterm baby boy born at 36 weeks of gestation of a twin pregnancy (diamniotic, dichorionic) via vacuum-assisted vaginal delivery was born to a 29-year-old primigravida who had prolonged rupture of membranes (PROM) lasting 72 hours associated with chorioamnionitis and intrapartum fever. Group B *Streptococcus *(GBS) status was declared negative, and appropriate antibiotics were given intrapartum. Amniotic fluid was meconium stained, and APGAR scores were 4 and 10 at the first and fifth minutes, respectively. In the delivery room, positive pressure ventilation was administered for one minute and supported with free-flow oxygen.

On physical examination, the patient was found to be in respiratory distress with retractions (subcostal/intercostal), tachypnea of 60 beats/minute, and nasal flaring. The patient was admitted to the neonatal intensive care unit (NICU), where he was kept on nasal continuous positive airway pressure (CPAP) with an oxygen saturation of 95%.

The patient was started on ampicillin and gentamicin after a partial sepsis workup. After 24 hours of admission, the patient started to improve. As the respiratory distress improved, CPAP was discontinued. The blood culture was negative, and the patient was kept in the NICU to complete the course of antibiotics. On the 10th day of life, three days after the antibiotics course was completed, the patient spiked a fever and was noticed to be irritable and crying excessively. A full sepsis workup was done, and the patient was started on meropenem and vancomycin. The results of the sepsis workup are presented in Table 1.

**Table 1:** Laboratory results after the fever spike. CBC: complete blood count; CRP: C-reactive protein; CSF: cerebrospinal fluid; Hbg: hemoglobin; RBC: red blood cell; Hct: hematocrit; MCH: mean corpuscular hemoglobin; RDW: red cell distribution width; WBC: white blood cell; Lymph: lymphocytes; Seg: segments; Plt: platelets; Mono: monocytes; Eos: eosinophils, ESBL-producing *E. coli*: extended-spectrum beta-lactamases-producing *Escherichia coli*

Test	Results	Normal values
CBC	Hbg: 12.6 g/dL	15–21
	RBC: 3.31 mil/µL	4–6
	Hct: 37.1%	46–65.5
	MCH: 38.1 pg	24–34
	RDW: 16.4%	11.5–14.5
	WBC: 14.2 k/µl	5–21
	Lymph: 27%	41–48
	Seg: 53%	35–45
	Plt: 124 k/µL	140–450
CRP	19.49 mg/dL	0.1–0.5
Procalcitonin	13.81 ng/mL	≤0.1
CSF	Protein: 556 mg/dL	15–40
	Glucose: <5 mg/dL (serum glucose: 85 mg/dL)	60–80
	Appearance: yellow/turbid	Clear
	RBC: 800 mm^3^	≤1,000
	WBC: 5,076 mm^3^	0–30
	Seg: 95%	≤4
	Mono: 4%	50–90
	Eos: 1%	≤0
	Gram stain: no organism seen	No organism
Urine analysis	Color: amber	Yellow
	Clarity: clear	Clear
	pH: 7	5.5–6
	Nitrite: negative	Negative
	WBC: 2–5	0–2
	RBC: 0–2	0–3
	Bacteria: 3+	None
Cultures	Blood culture: ESBL-producing E. coli	No growth
	Urine culture: no growth	No growth
	CSF culture: no growth	Aerobic culture: no growth in 48 hours

Blood culture was sensitive to amikacin, imipenem, and meropenem and resistant to ciprofloxacin, gentamicin, levofloxacin, and trimethoprim/sulfamethoxazole. Results indicated a partially treated bacterial meningitis with a blood culture positive for ESBL.

Computed tomography (CT) of the head was done and was unremarkable as it showed homogenous density with preserved gray-white matter differentiation. Repeated blood culture after three days was negative. Vancomycin was discontinued as the blood culture and cerebrospinal fluid (CSF) culture were both negative for 48 hours. The patient was discharged in good health after receiving 14 days of meropenem.

The patient presented to the emergency room 17 days later with a history of fever of 38.5°C associated with vomiting, decreased activity, decreased feeding with poor sucking, irritability, and excessive crying. The patient’s physical examination was unremarkable, apart from being febrile and having adequate growth since birth. The patient was admitted to the pediatric ward with a case of fever to rule out sepsis. Routine labs were sent as well as a full sepsis workup and the patient was started on meropenem and vancomycin. The laboratory results are presented in Table 2.

**Table 2:** Laboratory results during the second presentation. CBC: complete blood count; CRP: C-reactive protein; CSF: cerebrospinal fluid; Hbg: hemoglobin; RBC: red blood cell; Hct: hematocrit; MCH: mean corpuscular hemoglobin; RDW: red cell distribution width; WBC: white blood cell; Lymph: lymphocytes; Seg: segments; Plt: platelets; Mono: monocytes; Eos: eosinophils

Test	Results	Normal values
CBC	Hbg: 8.6 g/dL	15–21
	RBC: 2.56 mil/µL	4–6
	Hct: 25%	46–65.5
	MCH: 98 pg	24–34
	RDW: 16.4%	11.5–14.5
	WBC: 17.5 k/µl	5–21
	Lymph: 28%	41–48
	Seg: 64%	35–45
	Plt: 597 k/uL	140–450
CSF	Protein: 361 mg/dL	15–40
	Glucose: 1 mg/dL (serum glucose: 81 mg/dL)	60–80
	Appearance: turbid	Clear
	RBC: 1,440 mm^3^	≤1,000
	WBC: 8,200 mm^3^	0–30
	Seg: 84%	≤4
	Mono: 5%	50–90
	Gram stain: no organism seen	No organism
	Aerobic culture: no growth in 24 hours	No growth
CRP	1.05 mg/dL	0.1–0.5
Meningitis/Encephalitis molecular ID	E. coli K1	No growth
Blood culture	No growth in 24 hours	No growth

Results indicated bacterial meningitis with a molecular culture of the CSF sample showing the presence of *E. coli* K1, for which the patient was shifted to meropenem and amikacin. Urine analysis, liver function test, renal function test, erythrocyte sedimentation rate, procalcitonin, ammonia, and lactic acid were normal. A chest X-ray was done and was unremarkable.

During admission, the patient had a spike in fever reaching 38°C. Furthermore, the patient was noticed to have jitteriness and lip-smacking that was distractible.

**Figure 1:**
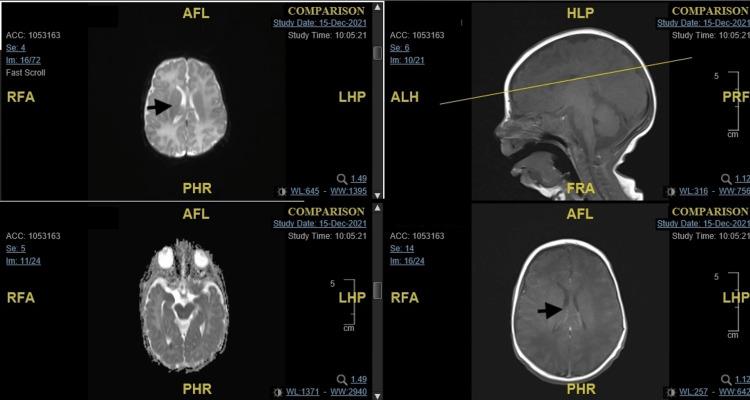
Magnetic resonance imaging showing ventricular enlargement with no signs of meningitis.

Magnetic resonance imaging (MRI) and electroencephalogram (EEG) were done. MRI showed a focus of diffusion restriction within the dependent portion of the right lateral ventricle with no evidence of pathological enhancement as well as ependymal or meningeal pathological enhancement, suggestive of debris as a sequela of prior meningitis (Figure 1). EEG was within normal limits for age. As EEG was reassuring, no epilepsy medications were started.

No further abnormal movements were noticed, and the patient received amikacin for 14 days until the lumbar puncture was repeated and turned out negative. Moreover, the patient received meropenem for a total of 56 days as a treatment for relapse and was discharged with good health, and follow-ups were done at the ID clinic.

The patient presented to his two follow-ups in the second and seventh months. He was doing well, no abnormal movements were noticed, no episodes of fever were present, and developmental milestones were appropriate for his age.

## Discussion

Neonatal meningitis is defined as meningeal inflammation during the first 28 days of life. It can be classified as early-onset meningitis, presenting in the first seven days of life, or late-onset meningitis, presenting between eight and 29 days postnatally. Furthermore, bacterial meningitis is most commonly caused by GBS (50% of cases), followed by *E. coli* (20% of cases) [5]. The incidence of bacterial meningitis due to GBS has declined over the years through screening and intrapartum antibiotic prophylaxis; however, gram-negative organisms such as *E. coli* and *Klebsiella* may be more common in developing countries [6].

Neonates with meningitis may present with unstable temperature, neurological findings (including irritability, lethargy, hypotonia, or seizures), poor feeding or vomiting, decreased activity, and respiratory distress.

Characteristic lab results of neonatal meningitis are abnormal peripheral blood counts, abnormal CSF findings (including elevated WBC and protein, as well as decreased glucose), and a positive culture or gram stain from the CSF or positive blood culture, as both will be positive for the same organism. However, CSF culture can be negative in up to one-third of cases, making the blood culture the only positive test in some cases [5,6].

In the first presentation, our patient was febrile and irritable, and in the second presentation, the patient was febrile with poor feeding and decreased activity. In both presentations, the patient had significant findings in the CSF; however, CSF culture and gram stain were negative in both presentations, and blood culture was positive in the first, while the CSF molecular ID was positive in the second.

A widespread ESBL-producing *E. coli* has been noticed in hospitals worldwide. ESBL are enzymes that cause resistance to beta-lactam antibiotics (penicillin, cephalosporins, and aztreonam); however, carbapenems are considered the most resistant to degradation by ESBL. ESBL-producing organisms are mostly found in gram-negative organisms, mainly in *Klebsiella pneumoniae*, *Klebsiella oxytoca*, and *E. coli*. The main reservoir for ESBL-producing *E. coli* is in the gastrointestinal tract, which is seen more in patients who had healthcare exposure such as hospitalization. Other risk factors include prior administration of antibiotics, ICU stay, immunocompromised patients, and the placement of catheters [7]. The patient’s maternal risk factors (PROM, primigravida, fever during delivery, respiratory distress, meconium staining), along with the initial use of antibiotics and his stay in the NICU, made him at great risk of ESBL-producing *E. coli*.

Treatment differs as ESBL has different varieties, and each variant has its own activity against beta-lactam substrates; however, the main choices of treatment in these cases are carbapenem (imipenem, meropenem, and ertapenem). In our patient, ESBL-producing *E. coli* was sensitive to amikacin, imipenem, and meropenem and was resistant to ciprofloxacin, gentamicin, levofloxacin, and trimethoprim/sulfamethoxazole. Furthermore, according to research, these organisms have higher mortality rates, require prolonged hospital stays and expenses, as well as increased resistance to microbiological agents compared to the typical *E. coli *[7].

In a previously reported case study by Puvabanditsin et al., in 2019, one case of ESBL-producing *E. coli *causing neonatal meningitis was reported in a 12-day-old baby girl who had some similar risk factors to our patient (PROM, preterm, chorioamnionitis, and NICU admission). The case was complicated by a cerebral abscess and treated with meropenem for nine weeks and amikacin for six weeks [8].

Another reported case series reported by Moissenet et al., in 2010, highlighted an outbreak of ESBL-producing *E.coli* that occurred in the neonatal wards back in 2009; 26 neonates out of 59 who were screened for ESBL were positive, and only one infant developed meningitis who was a term baby born via cesarean section. He did not receive any antibiotics; however, on his fifth day of life, he tested positive with the screening test and started to develop symptoms on the 10th day of life. They were treated with imipenem combined with gentamicin and ciprofloxacin [9].

According to the literature, 80 different capsular polysaccharide K antigens are produced by *E. coli*; the K1 strain, in particular, was studied extensively. Similar to group B *Neisseria meningitides* polysaccharide antigens, *E. coli *K 1 is an α-2,8-linked linear homopolymer of N-acetylneuraminic. According to research, K1 is the strain of *E. coli *that is shown to be particularly linked to meningitis, bacteremia, and septicemia in neonates [10]. Research showed that 80% of *E. coli* causing meningitis in the neonatal period is due to K1 strain as the capsule of K1 is able to cross the blood-brain barrier [4].

Previously reported cases of neonatal meningitis due to *E. coli K1* were of patients who were initially infected with the same pathogen of *E. coli* K1, and in these reported cases, one of the patients was treated with appropriate antibiotic coverage of gentamicin and systemic cotrimoxazole [11,12].

To our knowledge, no cases were reported of neonates who got meningitis due to ESBL-producing *E. coli*, with a relapse of *E. coli* K1.

Despite the fact that previously reported cases of *E. coli* K1 relapse involved patients who were initially diagnosed with the same organism, our case report differed from them in that it did not follow the previously reported patterns as the *E. coli *K1 meningitis was followed after the ESBL-producing *E. coli*, with the reassurance and confirmation from the microbiology team denying possible contamination in the sample.

## Conclusions

ESBL-producing *E. coli *has started to emerge recently, and meningitis due to ESBL-producing *E. coli* is highly uncommon. In patients who are at high risk, the possibility of developing ESBL-producing *E. coli *should be taken into account. Although *E. coli *K1 was reported to cause relapse in patients who were initially infected with it, it can also cause relapse in other types of *E. coli*. With early detection and proper management, complications of the two types can be avoided.
